# P-2101. Coverage Of The Molecular Diagnostic Technology For Pulmonary Tuberculosis In Bangladesh: Diagnostic Milestone For Control Of Tuberculosis

**DOI:** 10.1093/ofid/ofae631.2257

**Published:** 2025-01-29

**Authors:** Ahmadul Hasan Khan, Shakila Yeasmin, Sanjida Anjum, F M Monirul Haque

**Affiliations:** National Tuberculosis Control Program, Directorate General of Health Services, Dhaka, Bangladesh, Dhaka, Dhaka, Bangladesh; National Tuberculosis Control Program, Directorate General of Health Services, Mycobacterial Disease Control (MBDC), Dhaka, Bangladesh, Dhaka, Dhaka, Bangladesh; National Tuberculosis Control Program, Directorate General of Health Services, Dhaka, Bangladesh, Dhaka, Dhaka, Bangladesh; National Tuberculosis Control Program, Directorate General of Health Services, Dhaka, Dhaka, Bangladesh

## Abstract

**Background:**

Provision of early and accurate Tuberculosis (TB) diagnosis is crucial to end TB specially in a densely populated like Bangladesh. Molecular WHO-approved rapid diagnostic tests for TB (mWRDs) can significantly improve identification of undiagnosed TB cases. This study estimates the coverage of mWRDs among the total detected Pulmonary TB (PTB).

**Figure:**
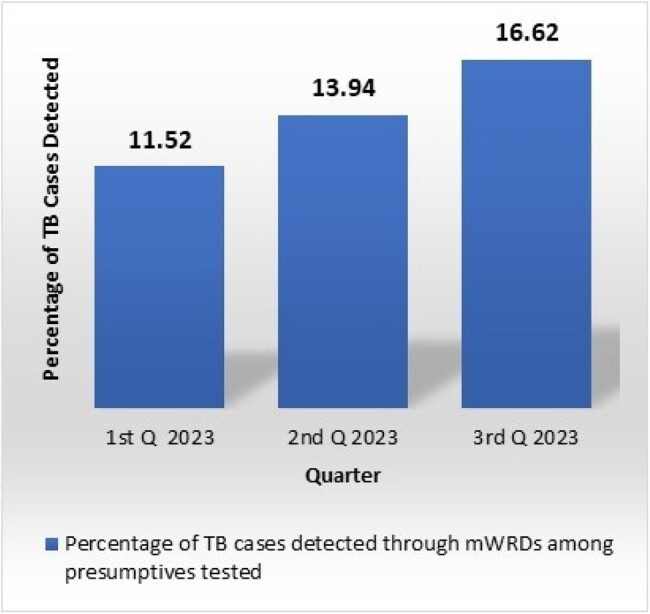

**Methods:**

Monthly programmatic data on nationwide TB diagnosis in Bangladesh using mWRDs were pulled and compiled. Routine programmatic data for TB detection using mWRDs were segregated and compared quarterly for the first three quarter of 2023 to know the percentage of molecular testing coverage among presumptive TB tested and percentage of pulmonary TB notified diagnosed through molecular testing during that time.

**Figure:**
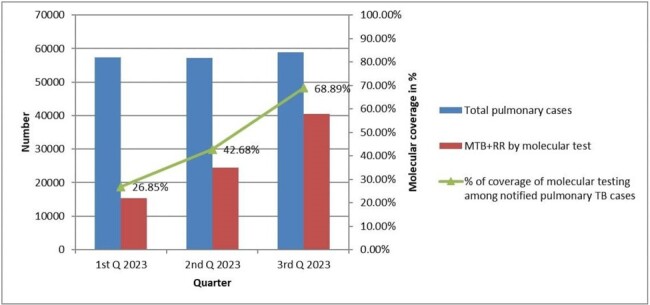

**Results:**

A total of 5,53,172 presumptive cases undergone mWRDs to detect the *Mycobacterium Tuberculosis* (MTB) and Rifampicin Resistant (RR) cases. Among them, 80,414 (14.54%) cases were detected as MTB including RR. During the 1st, 2nd, and 3rd quarters of 2023, the total number of presumptive tests conducted were 133,845, 175,038, and 244,289, respectively. Among them, the detected TB cases according to each quarter were 15,418 (11.52%), 24,397 (13.94%), and 40,599 (16.62%), respectively (Figure 1). With this data it was obvious that in the 3^rd^ quarter of 2023 the percentage of case detection by mWRDs increased compared to the consecutive previous two quarters. It revealed that, percentage of case detection by mWRDs gradually increased over the time during 1^st^ to 3^rd^ quarter 2023. To assess the coverage of mWRDs, quarterly data was analyzed and compared. It was determined how many TB cases were confirmed using molecular technology (GeneXpert and TrueNat) out of the total number of pulmonary cases over time. The analysis revealed that the quarterly percentage coverage of mWRDs was 26.85%, 42.68%, and 68.89% during the 1st, 2nd, and 3rd quarters, respectively, indicating an increasing trend over time (Figure 2).

**Conclusion:**

Scaling up the mWRDs as a point of care diagnostic test for presumptive TB cases in resource limited settings would have a significant impact to meet the SDG and End TB strategy for TB detection. Increasing coverage of molecular testing for more PTB case detection is inevitable considering the context of Bangladesh.

**Disclosures:**

All Authors: No reported disclosures

